# Factors associated with subjective oral health among older adults aged 65 and over living alone: the role of social capital

**DOI:** 10.1186/s12889-025-22649-9

**Published:** 2025-04-15

**Authors:** Eun-Ju Park, Yoon Min Gil

**Affiliations:** 1 Dental Research Institute, Seoul National University, 101 Daehak-Ro, Jongno-Gu, Seoul, South Korea; 2 Dental Research Institute, School of Dentistry, Seoul National University, 101 Daehak-Ro, Jongno-Gu, Seoul, South Korea

**Keywords:** Social capital, Subjective Oral health, Older adults living alone, Korea Community Health Survey

## Abstract

**Background:**

With the global increase in the number of older adults and single-person households, attention is increasingly being directed toward older adults living alone as a vulnerable population in public health. These individuals are particularly susceptible to deficiencies in social capital, one of the key social determinants of health. However, research on the relationship between social capital and oral health among older adults living alone remains limited. Therefore, this study aims to analyze the factors associated with oral health in this population, with a specific focus on social capital.

**Methods:**

This study analyzed data from the Korean Community Health Survey, 2023, focusing on 21,677 older adults aged 65 and over living alone. The dependent variable was subjective oral health level, while independent variables included social capital factors such as trust, reciprocity, social network, and social participation. Hierarchical logistic regression analysis was conducted to investigate the relationship between social capital and subjective oral health levels, with results presented as odds ratios and 95% confidence intervals.

**Results:**

Older adults aged 65 and over living alone were found to be particularly vulnerable to poor subjective oral health, especially those with older age, lower educational attainment, lower income levels, lack of economic activity, and residence in rural areas. Among the social capital variables, the analysis showed that individuals without reciprocity had 1.30 times higher odds of worse subjective oral health compared to those with reciprocity (OR = 1.30, 95% CI 1.18–1.42). Similarly, individuals without social participation had 1.31 times higher odds of worse subjective oral health compared to those with social participation (OR = 1.31, 95% CI 1.21–1.42).

**Conclusions:**

Social capital is significantly associated with the subjective oral health levels of older adults aged 65 and over living alone. This emphasizes the importance of enhancing social capital to mitigate oral health inequalities among socially vulnerable older adults living alone.

## Background

Social capital refers to resources that can be obtained through social networks at the individual and community levels, thereby enhancing the efficiency of society [1, 2]. Social capital can be categorized into structural and cognitive social capital [3, 4]. Structural social capital refers to the structure of social networks and patterns of community participation, including objectively observable networks, institutions, rules, and alliances [3–5]. In contrast, cognitive social capital consists of subjective elements such as trust in others, reciprocity, norms, values, and attitudes. Each type influences health outcomes through distinct pathways [3–5]. Social capital is recognized as a social determinant of health, influencing both health outcomes and health-related behaviours [5–7]. Insufficient social capital has been associated with an increased risk of various health conditions, including cardiovascular diseases [8–12], chronic obstructive pulmonary disease [13, 14], cerebrovascular diseases [15], Alzheimer’s disease [16], and mental health disorders such as depression, anxiety, stress, and post-traumatic stress disorder [17–19]. Furthermore, low levels of social capital have a detrimental impact on overall health, comparable to risk factors such as smoking and alcohol consumption [20, 21], and contribute to higher mortality rates [22, 23].

Single-person households and older adults are particularly vulnerable to insufficient social capital [24, 25]. Single-person households experience heightened challenges in areas including social relationships, health, safety, housing, and financial stability when compared to multi-person households [24–26]. Similarly, older adults are at heightened risk of social isolation due to diminished social networks, often resulting from the loss of family and friends, retirement, and reduced mobility [27, 28]. Single-person households have shown consistent growth, with EUROSTAT reporting a 21.0% increase in single-person households in the European Union from 2013 to 2023 [29], and the U.S. Census indicating that single-person households accounted for 29% of all households in the United States as of 2024 [30]. Notably, South Korea has experienced a significant rise in the proportion of single-person households, reaching 35.5% in 2023 [31]. Although variations exist across countries, the increase in single-person households is a global trend [32]. Along with the growing number of older adults, these groups are increasingly recognized as vulnerable populations with limited social capital. This trend is emerging as a significant global public health issue, underscoring the urgent need for increased attention and support to enhance the social capital of these groups [33, 34]. While these demographic trends highlight growing vulnerabilities, the intersection of social capital and health outcomes in these populations remains underexplored—particularly in the context of oral health.

This gap extends to oral health—a critical yet often overlooked dimension of overall well-being. Social capital is also recognized as an important social determinant of oral health. Previous studies have demonstrated that insufficient social capital increases the risk of dental caries, traumatic injuries, and periodontal diseases [35–38]. Social capital influences oral health behaviors through social norms, informal social control, and the acquisition of health knowledge. Additionally, social support from family and friend networks provides a sense of security and belonging, which alleviates stress and reduces susceptibility to oral diseases. Moreover, the dissemination and sharing of health information enhances access to dental care services, thereby promoting overall oral health. However, current studies predominantly examine general older adult population or younger single-person households, neglecting the unique challenges faced by older adults living alone who experience cumulative disadvantages from both aging and social isolation [35, 37]. Therefore, there remains a significant gap in understanding the relationship between social capital and oral health specifically among older adults living alone, a group that represents a particularly vulnerable population due to the compounded effects of aging and social isolation.

The purpose of this study is to examine the association between social capital factors and oral health among older adults aged 65 and over living alone. To provide empirical evidence, we assessed the distribution of social capital related to subjective oral health in this population and employed a hierarchical model to elucidate the relationship between social capital factors and oral health. This investigation will extend social capital theory by examining its multiplicative effects in a doubly vulnerable population—those experiencing both advanced age and solitary living.

## Methods

### Data resource

This study is based on a cross-sectional survey using raw data from the 2023 Korea Community Health Survey (KCHS). The KCHS is an annual health survey conducted by the Korea Disease Control and Prevention Agency under the Local Health Act and its Enforcement Decree [39]. It targets adults aged 19 and older across 258 urban and rural districts nationwide. Sampling is done using the probability proportional system method, followed by system extraction to select households. Trained investigators visit households to conduct one-on-one interviews using questions developed for the Community Health Survey through CAPI (Computer-Assisted Personal Interviewing). The survey questions cover topics such as health behaviors, diseases, vaccinations, activity limitations, quality of life, healthcare utilization, accidents, personal hygiene, environment, education, and economic activities [37]. Respondents must be able to communicate directly, and individuals requiring a proxy for communication are excluded from the survey in accordance with the survey protocol.

### Study participants

The participants of this study were 21,677 individuals aged 65 and over living alone. In 2023, the KCHS included 231,752 adults aged 19 and over from 258 urban and rural districts. Among them, 81,898 individuals aged 65 and over were initially selected, and from this group, 21,677 individuals living alone were chosen as the final study participants, defined as older adults living alone.

### Dependent variable

The dependent variable in this study was subjective oral health status. Participants responded to the question, "How would you rate the condition of your oral health, including your teeth and gums?" using a 5-point Likert scale: very poor, poor, fair, good, and very good. Responses of "poor" and "very poor" were reclassified as "poor," while those of "fair," "good," and "very good" were reclassified as "good." Previous studies have suggested that subjective oral health status represents a multidimensional approach to assessing oral health, incorporating individuals' social and psychological dimensions [40]. Furthermore, as their subjective assessments can influence treatment decisions, it has recently been recognized as an important indicator for evaluating oral health conditions [41, 42].

### Independent variables

As an independent variable, social capital was composed of trust, reciprocity, social network, and social participation. Trust, a commonly used indicator for measuring social capital, is known to be associated with health [43]. In this study, trust was measured using responses to the question: "Do people in your neighborhood trust and rely on each other?" with answers dichotomized as either "yes" or "no." Reciprocity was assessed using responses to the question: "Do people in your neighborhood help and support each other during important life events such as weddings and funerals?" with binary options of "yes" or "no." Individuals who experience failures in reciprocity, which is rooted in mutual support and exchange, are more likely to be exposed to health risks and diseases [44]. Social network was evaluated through the question: "How often do you meet or contact your close relatives (including family), neighbors, or friends?" Responses were dichotomized into "less than once a month" and "once a month or more," which were further classified as "absent" or "present." Social networks are known to prevent social isolation and reduce the risk of diseases [10]. Social participation was determined based on responses to the question: "Do you regularly participate in any of the following activities at least once a month: religious activities, social gatherings, leisure/recreational activities, or volunteer activities?" Participants who answered "yes" to at least one activity were considered socially active. Social participation is regarded as a significant factor within social capital that contributes to health improvement [45].

### Control variables

Control variables in this study included sociodemographic variables and oral health behaviour variables.

Sociodemographic variables encompassed sex, age, education level, income, economic activity status, and region. Age was categorized into two groups: 65–74 years as the "early older adults" and 75 years and older as the "late older adults." This classification was based on differences in health status, health behaviours, and social capital observed between these two age groups [46]. Education was divided into five levels: no education, elementary school, middle school, high school, and university or higher. Consistent with previous studies, a significant proportion of older adults in rural areas in Korea are uneducated, prompting a distinction between the "no education" group and those who completed elementary school [47]. Income was defined as each individual's monthly income. The raw data from the 2023 KCHS provided household income, which was converted to individual income using the OECD square root equivalence method [48]. Regions were classified into urban and rural areas, as rural areas were noted to have lower accessibility to healthcare services [49].

Oral health behaviour variables included toothbrushing practices, unmet dental care needs, and oral examinations. To assess toothbrushing practices, participants were asked: "Did you brush your teeth after lunch, or after dinner or before going to bed yesterday?" Responses were classified as "yes" or "no" based on whether participants reported brushing their teeth the previous day. Unmet dental care needs were evaluated using the question: "In the past year, have you needed dental care but been unable to receive it?" Responses were categorized as "yes" or "no." Oral examinations were assessed by asking: "In the past year, have you had an oral examination to assess your oral health even though you had no specific oral health issues?" Responses were similarly categorized as "yes" or "no." Previous studies have demonstrated that oral health behaviours significantly influence subjective oral health [50, 51].

### Statistical analysis

The KCHS employs a complex sampling design. Accordingly, this study applied a complex survey analysis approach, accounting for sampling weights, stratification variables, and cluster variables in all statistical analyses.

The general characteristics of the participants, older adults aged 65 and over living alone, were analyzed using frequency analysis. To examine differences in the distribution of subjective oral health levels across the study population, Rao-Scott chi-square test were performed on control and independent variables. The results are presented as F-Statistic and *P*-values. To investigate the influence of social capital on subjective oral health levels, hierarchical logistic regression analysis was employed. Model 1 included sociodemographic variables to evaluate their association with subjective oral health levels. Model 2 expanded on this by incorporating oral health behaviour variables alongside sociodemographic variables. Model 3 assessed the relationship between social capital and subjective oral health levels while controlling for both sociodemographic and oral health behaviour variables. The odds ratios (OR) and 95% confidence intervals (CI) were reported for each model. All statistical analyses were conducted using SAS version 9.4.

## Results

Table 1 presents the general characteristics of the study participants (*N* = 21,677). The majority were women (71.8%) and aged 75 years or older (52.6%). Education levels were generally low, with 57.4% having completed elementary school or less, including 26.3% with no education. More than half of the participants (57.4%) reported a monthly income of less than 1 million KRW, and 70.3% were not economically active. Most participants (68.6%) resided in urban areas. Regarding oral health behaviours, 93.7% practiced toothbrushing, but 64.6% reported not having undergone an oral examination in the past year. Additionally, 19.2% experienced unmet dental care needs. Subjective oral health levels were almost evenly distributed, with 51.2% rating their oral health as "bad." In terms of social capital, 69.0% of participants reported trust in their community, while 54.0% indicated no reciprocity in mutual help. Most participants (95.1%) maintained close social network, and 61.1% engaged in social participation.

**Table 1:** General characteristics of study participants (*N* = 21,677)

Variables	Categories	Unweighted N	Weighted %
Sex	Male	5,301	28.2
	Female	16.376	71.8
Age (years)	65–74	8,763	47.4
	≥ 75	12,914	52.6
Education	No education	7,956	26.3
	Elementary school	6,905	31.1
	Middle school	2,760	16.2
	High school	2,845	18.3
	University or higher	1,194	8.0
Income (month, million KRW)	< 1	14,171	57.4
	1—2	5,113	27.6
	2—3	1,533	9.6
	3—4	486	3.2
	≥ 4	349	2.2
Economic activity	Yes	8,045	29.7
	No	13,631	70.3
Region	Urban	8,324	68.6
	Rural	13,353	31.4
Toothbrushing practice	Yes	19,919	93.7
	No	1,754	6.3
Unmet dental care needs	No	14,718	80.8
	Yes	3,584	19.2
Oral examination	Yes	6,156	35.4
	No	15,511	64.6
Trust	Yes	16,530	69.0
	No	4,624	31.0
Reciprocity	Yes	13,975	46.0
	No	7,390	54.0
Social network	Yes	21,026	95.1
	No	651	4.9
Social participation	Yes	12,426	61.1
	No	9,251	39.8
Subject oral health levels	Good	9,824	48.8
	Bad	11,858	51.2

Table 2 presents the distribution of subjective oral health levels among older adults aged 65 and over living alone. A significant association was observed between education level and subjective oral health, with poorer oral health more prevalent among participants with lower education levels: 65.4% of those with no education reported poor oral health compared to 33.0% of those with a university degree or higher (*p* < 0.0001). Similarly, poorer oral health was more common in participants with lower income levels (60.1% for those earning less than 1 million KRW per month; *p* < 0.0001) and those who were non-economically active (46.1%) or resided in rural areas (56.1%; *p* < 0.0001). Regarding oral health behaviours, 68.6% of participants who did not brush their teeth the previous day and 73.2% of those with unmet dental care needs in the past year reported poor oral health (*p* < 0.0001). Additionally, 56.4% of participants who had not undergone an oral examination in the past year reported poor oral health, compared to 41.8% of those who had (*p* < 0.0001).

**Table 2:** Differences in subjective oral health levels across sociodemographic, social capital, and oral health behavioural factors

Variables	Categories	Subjective oral health levels		F	*p*-value
		Bad Weighted %	Good Weighted %		
Sex	Male	50.5	50.0	3.652	0.056
	Female	51.7	48.3		
Age (years)	65–74	44.6	55.4	245.962	< .0001
	≥ 75	57.2	42.8		
Education	No education	65.4	34.6	162.107	< .0001
	Elementary school	53.8	46.2		
	Middle school	45.5	54.5		
	High school	39.6	60.4		
	University or higher	33.0	67.0		
Income (month, million KRW)	< 1	60.1	39.9	175.896	< .0001
	1—2	43.1	56.9		
	2—3	34.1	65.9		
	3—4	29.8	70.2		
	≥ 4	29.8	70.2		
Economic activity	Yes	44.9	55.1	120.537	< .0001
	No	46.1	53.9		
Region	Urban	49.0	51.0	86.781	< .0001
	Rural	56.1	43.9		
Toothbrushing practice	Yes	50.1	49.9	145.567	< .0001
	No	68.6	31.4		
Unmet dental care needs	No	48.0	52.0	530.722	< .0001
	Yes	73.2	26.8		
Oral examination	Yes	41.8	58.2	296.456	< .0001
	No	56.4	43.6		
Trust	Yes	50.5	49.5	9.002	0.0027
	No	53.2	46.8		
Reciprocity	Yes	50.0	50.0	8.862	0.0029
	No	52.3	47.7		
Social network	Yes	50.6	49.4	45.364	< .0001
	No	63.6	36.4		
Social participation	Yes No	45.5 60.3	54.5 39.7	328.248	< .0001

All F-statistics and *p*-values were calculated using the Rao-Scott chi-square test

Table 3 presents the results of hierarchical logistic regression analysis to identify factors influencing subjective oral health status among older adults living alone. In Model 1, which included sociodemographic variables, education level and economic activity showed significant associations with subjective oral health. Participants with no education had the highest odds of poor subjective oral health (OR 2.84, 95% CI 2.41–3.35), and those who were economically inactive also had significantly higher odds (OR 1.18, 95% CI 1.10–1.27). Model 2 incorporated oral health behaviour variables in addition to sociodemographic variables. Among oral health behaviours, unmet dental care needs significantly increased the odds of poor subjective oral health (OR 2.62, 95% CI 2.37–2.90), followed by not brushing teeth (OR 1.69, 95% CI 1.44–1.98). Model 3 further included social capital variables while controlling for sociodemographic and oral health behaviour variables. Within social capital, participants without reciprocity were more likely to report poor subjective oral health (OR 1.30, 95% CI 1.18–1.42) compared to those with reciprocity. Similarly, participants without social participation had significantly higher odds of poor subjective oral health (OR 1.31, 95% CI 1.21–1.42).

**Table 3:** Hierarchical logistic regression analysis of factors influencing subjective oral health levels

Variables		Categories	Model 1^a^ OR (95% CI)	Model 2^b^ OR (95% CI)	Model 3^c^ OR (95% CI)
Sociodemographic	Sex	Male	1.00	1.00	1.00
		Female	0.72(0.67–0.78)	0.74(0.68–0.81)	0.79(0.72–0.86)
	Age (years)	65–74	1.00	1.00	1.00
		≥ 75	1.16(1.08–1.25)	1.19(1.10–1.29)	1.21(1.11–1.32)
	Education	University or higher	1.00	1.000	1.000
		High school	1.24(1.06–1.46)	1.23(1.04–1.46)	1.22(1.03–1.45)
		Middle school	1.51(1.29–1.77)	1.53(1.29–1.81)	1.51(1.27–1.79)
		Elementary school	1.98(1.69–2.31)	1.93(1.64–2.28)	1.95(1.65–2.31)
		No education	2.84(2.41–3.35)	2.69(2.25–3.21)	2.64(2.20–3.17)
	Income	≥ 4	1.00	1.00	1.00
	(month, million KRW)	3—4	0.96(0.71–1.31)	0.96(0.71–1.31)	1.09(0.77–1.54)
		2—3	1.12(0.86–1.47)	1.12(0.86–1.47)	1.19(0.88–1.61)
		1—2	1.38(1.06–1.78)	1.38(1.06–1.78)	1.47(1.09–1.97)
		< 1	2.23(1.72–2.88)	2.23(1.72–2.88)	2.14(1.60–2.87)
	Economic activity	Yes	1.00	1.00	1.00
		No	1.18(1.10–1.27)	1.17(1.08–1.26)	1.12(1.03–1.21)
	Region	Urban	1.00	1.00	1.00
		Rural	1.04(0.98–1.11)	1.02(0.95–1.09)	1.12(1.03–1.21)
Oral health behaviour	Toothbrushing practice	Yes		1.00	1.00
		No		1.69(1.44–1.98)	1.61(1.38–1.88)
	Unmet dental care needs	No		1.00	1.00
		Yes		2.62(2.37–2.90)	2.53(2.29–2.81)
	Oral examination	Yes		1.00	1.00
		No		1.30(1.21–1.41)	1.28(1.18–1.39)
Social capital	Trust	Yes			1.00
		No			1.03(0.93–1.13)
	Reciprocity	Yes			1.00
		No			1.30(1.18–1.42)
	Social network	Yes			1.000
		No			1.12(0.92–1.36)
	Social participation	Yes			1.00
		No			1.31(1.21–1.42)
	AIC		2730471.0	2267090.2	2173751.7
	-2 Log Likelihood		2730445.0	2267058.2	2173711.7
	McFadden R^2^		0.053	0.086	0.092
	Likelihood Ratio Test		< .0001	< .0001	< .0001

^a^Model 1: adjusted for Sex, Age, Education, Income, Economic activity, Region

^b^Model 2: adjusted for Sex, Age, Education, Income, Economic activity, Region, Brushing practice, Unmet dental care needs, Oral examination

^c^Model 3: adjusted for Sex, Age, Education, Income, Economic activity, Region, Brushing practice, Unmet dental care needs, Oral examination, Trust, Reciprocity, Social network, Social Participation

## Discussion

This study is significant as it analyzes the relationship between the subjective oral health and social capital factors among older adults aged 65 and over living alone, a group facing dual vulnerabilities—aging and social isolation. Our findings suggest that, even after controlling for demographic and oral health behavior variables, the subjective oral health level of older adults living alone is associated with reciprocity and social participation.

As global aging accelerates and single-person households become more common, addressing oral health inequalities related to social capital in this underrepresented group is increasingly crucial. A study conducted among Chinese adults aged 50 years and over found that structural social capital was associated with a higher risk of edentulism, whereas cognitive social capital showed no significant association [52]. In contrast, our study found that both structural social capital (i.e., social participation) and cognitive social capital (i.e., reciprocity) were significantly associated with self-rated oral health. Additionally, research in the UK reported that higher levels of social support were linked to better self-rated oral health and improved oral health-related quality of life among older adults [53]. Collectively, these findings reinforce the broader, cross-cultural importance of social capital in maintaining oral health.

Among the four social capital variables—trust, reciprocity, social network, and social participation—that served as independent variables in this study, older adults aged 65 and over living alone without reciprocity had 1.30 times worse subjective oral health levels compared to those with reciprocity (OR 1.30, 95% CI: 1.18–1.42). Additionally, those who did not engage in social participation had 1.31 times worse subjective oral health levels than those who did (OR 1.31, 95% CI: 1.21–1.42). Although poorer subjective oral health was observed among those lacking trust and social network, these associations were not statistically significant. These findings highlight reciprocity and social participation as key social capital factors associated with subjective oral health among older adults living alone. This result aligns with previous studies that have shown that among the dimensions of social capital, both reciprocity and social participation are associated with subjective oral health levels [54, 55]. Reciprocity plays a vital role in transmitting behavioral norms within social networks. It facilitates the establishment and reinforcement of healthy norms, discourages aberrant social behaviors, fosters mutual support, safeguards vulnerable populations, and encourages health-promoting behaviors [3, 8, 43, 56]. Social participation enables individuals to access health-related resources through formal and informal networks. It facilitates the dissemination of health information, strengthens communication channels, and enhances social support, enabling individuals to acquire the necessary resources for maintaining and improving their health [4, 8, 57–60].

Regarding demographic variables, older age and lower education levels were associated with poorer subjective oral health. Additionally, the lower-income group exhibited worse subjective oral health compared to the higher-income group. Furthermore, the non-economically active group had poorer subjective oral health than the economically active group, and those living in rural areas reported worse subjective oral health than their urban counterparts. This highlights a significant association between the subjective oral health of older adults aged 65 and over living alone and their socioeconomic vulnerability. Previous studies have also reported that older adults living alone are more likely to be malnourished, experience poorer overall health, suffer from cognitive impairment, have limitations in daily activities, and display depressive symptoms compared to those living with others [20, 50].

The primary mechanisms through which social capital influences oral health include behavioral determinants, psychosocial factors, and access to oral healthcare services [3, 57]. Firstly, a key pathway through which social capital influences health is through behavioral factors [53]. Social capital can influence oral health behavior through social norms and informal social control, as well as the acquisition of health-related information and knowledge. For example, this includes smoking cessation, alcohol abstinence, vegetable consumption, regular toothbrushing, the use of oral hygiene adjuncts, and regular dental examinations. Secondly, regarding psychosocial factors, oral health is influenced by social networks with communities, friends, and family, as well as social participation. These factors help reduce stress by fostering a sense of stability and belonging [3]. Elevated stress levels trigger cortisol secretion, which can lead to immunosuppression, metabolic dysregulation, and an increased risk of periodontal disease and dental caries. In cases of periodontal disease or dental caries, stress-induced hormone secretion reduces immune resistance to bacteria, increasing susceptibility to oral diseases [3, 34, 35, 61]. Thirdly, regarding oral healthcare service factors, social participation can facilitate the acquisition of oral health knowledge, the adoption of health-promoting behaviors, and social support. This enhances the utilization of oral healthcare services and influences oral health outcomes [3].

According to the WHO's definition of health promotion in the Ottawa Charter (1986), health promotion is a process that enables individuals to take greater control of and improve their health. Within this framework, social capital plays a critical role in supporting health behaviors. It helps establish social norms, exerts informal social control, and facilitates the acquisition of health-related knowledge [3, 62]. Additionally, studies have shown that public health interventions grounded in social capital enhance social participation and promote health among older adults [36, 63, 64]. In contrast, social isolation has been associated with poorer oral health outcomes [35, 65]. Higher levels of social capital facilitate faster access to essential support, promote healthier behaviours, and strengthen the immune system [57, 60, 66, 67]. Given these findings, there is a need to focus on policies that strengthen social capital to improve the oral health of older adults living alone. Hikichi et al. (2015) [64] demonstrated that community-based intervention programs in Japan significantly reduced the incidence of functional disability among older adults by promoting social interactions. Building on this model, community-level interventions can serve as effective strategies for maintaining the health of older adults. Technology-based interventions also show potential for reducing social isolation [68]; thus, a blended approach combining face-to-face and technology-based programs may offer a valuable strategy for supporting the health of older adults living alone who are at high risk of social isolation.

The limitations of this study include the use of binary "yes/no" measurements for the social network and social participation variables within the social capital framework. These measurements do not capture detailed information such as specific targets, types, or intensity. This limitation in measurement could lead to a loss of information. For instance, larger social networks have been associated with a lower morbidity rate of periodontal disease [69, 70], highlighting the need for a more detailed assessment of social networks. Similarly, social participation measures could be enhanced by considering both the frequency of participation and the types of activities involved. These aspects should be explored in future research. Additionally, the cross-sectional design of the study constrains the capacity to infer causality. To address this limitation, future research should consider using longitudinal data to examine the long-term effects of social capital on oral health. Furthermore, intervention studies could be conducted to assess the effectiveness of social capital-based programs in improving oral health outcomes. Lastly, health-related variables, including chronic disease status and health-related behaviours, were not included as covariates, which represents a limitation of this study.

Nevertheless, this study offers several strengths. First, the data used in this study were sourced from the KCHS, a nationally representative 1:1 interview survey conducted by the Korea Disease Control and Prevention Agency, ensuring the validity and representativeness of the sample. Second, a hierarchical regression model was employed to examine the influence of social capital variables on subjective oral health while controlling for sociodemographic and oral health behaviour variables. Third, in the context of global aging and the increasing prevalence of single-person households, this study focused on older adults aged 65 and over living alone, a group particularly vulnerable to social isolation and disconnection. By addressing oral health inequalities related to social capital in this understudied population, this research provides significant value.

## Conclusions

This study identified significant associations between social capital factors—specifically reciprocity and social participation—and subjective oral health levels among older adults aged 65 and over living alone. These findings underscore the pivotal role of social capital in promoting oral health. They also highlight the need for targeted interventions to strengthen social capital as a means to address oral health inequalities, particularly among socially vulnerable older adult populations. Future research should explore more diverse dimensions of social capital to deepen our understanding of its impact on oral health.

## Data Availability

This dataset uses secondary data that has been processed with personal information, and it can be used after obtaining approval from the Korea Disease Control and Prevention Agency.
